# Association Between Salivary *Circular RNAs* Expression and Periodontal Disease Status

**DOI:** 10.1111/jre.70004

**Published:** 2025-07-01

**Authors:** Pingping Han, Kexin Jiao, Peter Mark Bartold, Andrew Liaw, Wei Wei, Sašo Ivanovski

**Affiliations:** ^1^ The University of Queensland, School of Dentistry, Epigenetics Nanodiagnostic and Therapeutic Group, Center for Orofacial Regeneration, Rehabilitation and Reconstruction (COR3) Brisbane Queensland Australia; ^2^ The University of Queensland, School of Dentistry Brisbane Queensland Australia; ^3^ Department of Neurosurgery Zhongnan Hospital of Wuhan University Wuhan China

**Keywords:** biomarker, circular RNA, epigenetics, periodontal diagnostics, salivary diagnosis

## Abstract

Salivary circular RNAs, particularly *hsa_circ_0003563 (circRUNX2)* and *hsa_circ_0001161 (circMMP9)*, show strong potential as non‐invasive biomarkers for diagnosing periodontitis and distinguishing the rate of disease progression, offering promising tools for improved periodontal diagnostics.

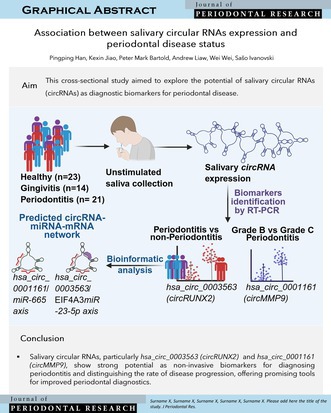

## 1 Introduction

Periodontitis is a chronic inflammatory disease driven by immune dysregulation and oral biofilm dysbiosis, leading to periodontal tissue destruction. Alongside genetics, epigenetic factors like bacteria, stress and lifestyle contribute to periodontitis. Traditional clinical diagnostics reflect past activity but not current disease status [1], highlighting the need for new saliva‐based epigenetic biomarkers for improved disease detection.

Saliva, containing epigenetic molecular markers like DNA methylation and non‐coding RNAs [2], offers a non‐invasive diagnostic tool. Circular RNAs (circRNAs) are stable, closed‐loop non‐coding RNAs that resist degradation, regulate gene expression [3], and are enriched in tissues and biofluids, linking them to various diseases [4]. Though studied in periodontitis tissues and cells [5], their specific salivary expression profiles in periodontal disease remain largely unexplored.

## 2 Methods

This cross‐sectional study (approved by Metro North Hospital and Health Service and University of Queensland ethics committees; approvals 54584 and 2018001225) randomly recruited participants from the Oral Health Centre (Dec 2018–Feb 2024). Due to the novelty of the topic, there was no prior data to inform the sample size. Based on classification guidelines [6], 23 healthy, 14 gingivitis, and 21 periodontitis were enrolled (Table S1). Inclusion criteria included age ≥ 18, ≥ 20 teeth, and no recent periodontal treatment, antibiotics, or long‐term anti‐inflammatories; individuals with systemic disease, pregnancy, or smoking history were excluded. Unstimulated saliva was collected pre‐examination for circRNA profiling. Ten circRNAs from protein‐coding genes linked to periodontitis—related to bone turnover (ALP, RUNX2), Wnt pathway (GSK3β, CTNNB, WNT5A), and matrix metalloproteinases (MMP9, MMP16, MMP17)–were selected using divergent primers designed via CircInteractome (Figure S1 and Table S2). Diagnostic performance of five differentially expressed *circRNAs* was assessed using ROC and AUC analysis. Group comparisons were conducted using the Kruskal‐Wallis test with Dunn's post hoc, and Mann–Whitney *U* tests for binary comparisons.

## 3 Results

As shown in Table S1, the study included 58 non‐smoking, systemically healthy participants (aged 22–88) of mixed gender and ethnicity, with no significant age differences between groups. BOP and PI were significantly higher in gingivitis and periodontitis than in healthy participants. Periodontitis patients showed increased average PPD (4.23 ± 1.32 mm) and deep pockets ≥ 5 mm (29 ± 19.18), consistent with Stage III/IV disease. All 21 had generalized periodontitis: 12 Grade B and 9 Grade C.

**FIGURE 1:**
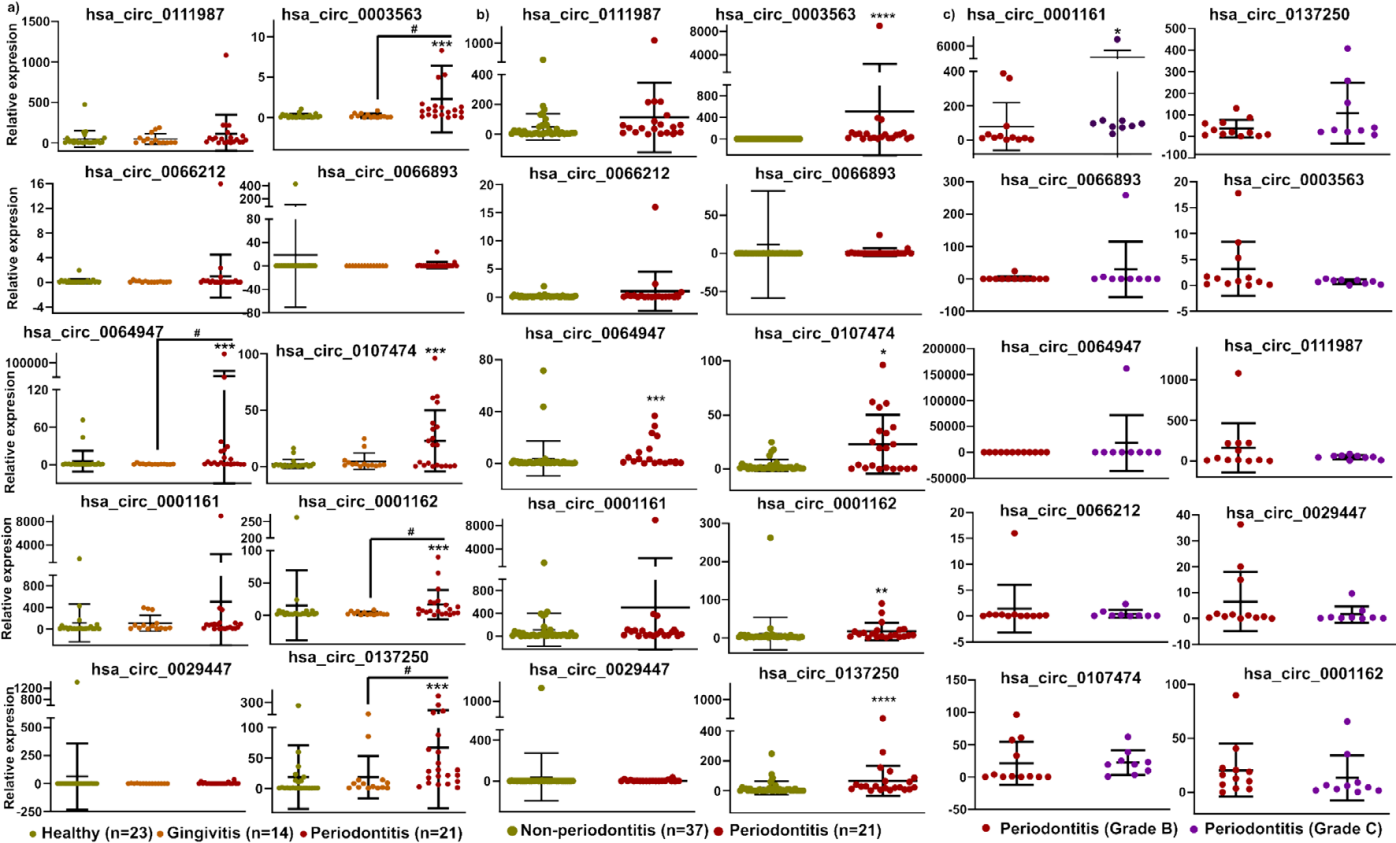
The relative expression of salivary *circRNAs* across different periodontal conditions. (a) Comparison among healthy, gingivitis, and periodontitis groups using one‐way ANOVA. ***p < 0.0002 for healthy versus periodontitis; #*p* < 0.05 for gingivitis versus periodontitis. (b) Differential expression of salivary *circRNAs* between non‐periodontitis (combining healthy and ginvitis) and periodontitis subjects assessed by the Mann–Whitney *U* test. *p < 0.03, **p < 0.002, ***p < 0.0002, ****p < 0.0001. (c) Comparison between Grade B and Grade C periodontitis patients using the Mann–Whitney *U* test; **p < 0.002 for Grade B versus Grade C.

Five circRNAs (*hsa_circ_0003563*, *hsa_circ_0064947*, *hsa_circ_0107474*, *hsa_circ_0001162*, and *hsa_circ_0137250*) were significantly upregulated in periodontitis compared to healthy controls, and four of these (excluding *hsa_circ_0107474*) were also significantly elevated compared to gingivitis patients (Figure 1a). The remaining five circRNAs showed no significant differences across groups. Grouping healthy and gingivitis as non‐periodontitis, these same five circRNAs significantly distinguished periodontitis (*n* = 21) from non‐periodontitis (*n* = 37) (Figure 1b). Additionally, *hsa_circ_0001161* showed a significant difference between Grade B (*n* = 12) and Grade C (*n* = 9) periodontitis patients (Figure 1c).

**FIGURE 2:**
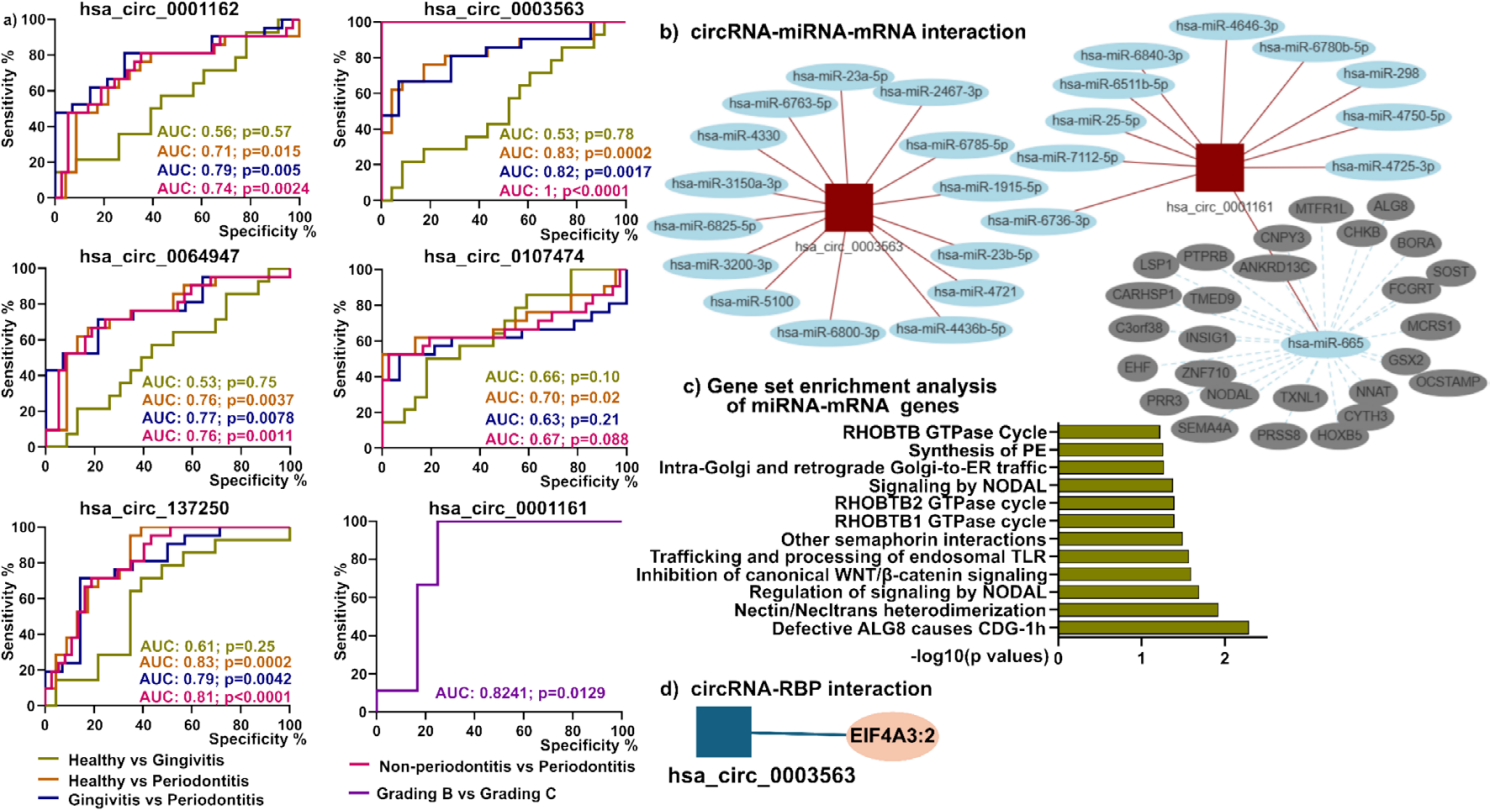
ROC Analysis, functional enrichment, and interaction networks of key circRNAs in the miRNA–mRNA–RBP Axis. (a) Receiver operating characteristic (ROC) curves and corresponding area under the curve (AUC) values for differentially expressed circRNAs. (b) Predicted miRNA‐mRNA networks and (c) Gene Ontology (GO) and Kyoto Encyclopedia of Genes and Genomes (KEGG) pathway enrichment analysis of predicted target genes associated with *hsa_circ_0003563 and hsa_circ_0001161*. (d) Integrated circRNA–miRNA–mRNA interaction networks and circRNA–RNA binding protein (RBP) interaction analysis by https://www.meb.ki.se/shiny/truvu/CircNetVis/.

The AUCs for gingivitis vs. healthy ranged from 0.53 to 0.67 (Figure 2a), while comparisons between periodontitis and healthy controls yielded higher AUCs: *hsa_circ_0001162* (0.71, *p* = 0.015), *hsa_circ_0064947* (0.76, *p* = 0.0037), *hsa_circ_0107474* (0.70, *p* = 0.02), and *hsa_circ_0137250* (0.83, *p* = 0.0002). Notably, *hsa_circ_0003563* showed the highest AUCs for periodontitis vs. healthy (0.82) and gingivitis vs. healthy (0.83) and achieved an AUC of 1.0 when distinguishing non‐periodontitis from periodontitis. For distinguishing between Grade B and Grade C periodontitis, *hsa_circ_0001161* yielded an AUC of 0.82 (*p* = 0.013), indicating its potential as a stratification marker.

CircRNA‐miRNA‐mRNA network analysis for *hsa_circ_0003563* and *hsa_circ_0001161* was performed using CircNetVis (Figure 2b) to predict miRNA targets and downstream mRNA pathways. A total of 26 miRNAs (e.g., hsa‐miR‐23‐5p) target *hsa_circ_0003563*, and 24 miRNAs (e.g., hsa‐miR‐665) target *hsa_circ_0001161*. Target genes of hsa‐miR‐665 were associated with pathways in inflammation (e.g., Nectin/Necl and WNT signalling), immune response (e.g., Endosomal TLR Trafficking, NODAL signalling), cellular traffic (e.g., Golgi‐ER Traffic, RHOBTB GTPase Cycle) and cellular dysfunction (e.g., ALG8 Deficiency in CDG‐1 h) (Figure 2c). EIF4A3 (Eukaryotic Translation Initiation Factor 4A3) was predicted to interact with *hsa_circ_0003563*, indicating a potential circRNA–RBP regulatory network (Figure 2d).

## 4 Discussion

A non‐invasive salivary test could serve as an useful adjunct diagnostic tool for detecting and monitoring periodontal disease, either as a chairside ‘point of care’ or at‐home test [7]. circRNAs like *hsa_circ_0084054* [5], *circMAP3K11*, *circCDK8* and *circCDR1* [8] have been shown to be upregulated in periodontitis tissues (reviewed in [9]). Salivary *hsa_circ_0001874* is a potential oral cancer biomarker [10], though its diagnostic value in periodontitis is unclear. Salivary circRNAs *hsa_circ_0003563* and *hsa_circ_0001161* emerged as biomarkers, distinguishing periodontitis from non‐periodontitis and Grade B from Grade C, respectively. Via validated axes like *hsa_circ_0084054/miR‐508‐3p*/PTEN [5, 8], our bioinformatic predictive model identified *hsa_circ_0003563/*EIF4A3*/miR‐23‐5p* and *hsa_circ_0001161/miR‐665* as potential regulators of immune response and inflammation in periodontitis. Future research should validate circRNA panels for early detection and monitoring of periodontitis, including post‐NSPT and surgical procedures. Integrating salivaomics and comparing saliva with gingival crevicular fluid [9] would advance understanding of pathogenic mechanisms, and support development of circRNA‐based point‐of‐care diagnostics, improving sensitivity and specificity for personalised periodontal care.

These findings suggest that salivary circRNAs could potentially complement clinical examinations as valuable screening tools, improving differentiation between periodontal health and disease. This research may advance salivary diagnostics and accelerate their clinical use for predicting periodontal status.

## Supporting information


Data S1.

## Data Availability

The data that support the findings of this study are available from the corresponding author upon reasonable request.
