# Supplementary material for: Association Between Salivary Circular RNAs Expression and Periodontal Disease Status
Source: J Periodontal Res. 2025 Jul 1;60(10):1053–5. doi: 10.1111/jre.70004 (PMC12640212; doi:10.1111/jre.70004)
Supplement: Supplementary file 1 — Data S1. [file JRE-60-1053-s001.docx]

**Supplementary information for**

**Association between salivary *circular RNAs* expression and periodontal disease status**

Pingping Han^1,2,*^, Kexin Jiao^1^, Peter Mark Bartold^2^, Andrew Liaw^1^, Wei Wei^3^, Sašo Ivanovski ^1, 2,*^

1. The University of Queensland, School of Dentistry, Epigenetics nanodiagnostic and therapeutic group, Center for Oral-facial Regeneration, Rehabilitation and Reconstruction (COR3), Brisbane, QLD 4006, Australia
2. The University of Queensland, School of Dentistry, Brisbane, QLD 4006, Australia
3. Department of Neurosurgery, Zhongnan Hospital of Wuhan University, 430000, Wuhan, China

*Correspondence: S.I.: [s.ivanovski@uq.edu.au](mailto:s.ivanovski@uq.edu.au); P.H.: [p.han@uq.edu.au](mailto:p.han@uq.edu.au)

**Materials and methods**

**Study participants**

This cross-sectional study was approved by the Human Ethics Committees from both the Metro North Hospital and Health Service and the University of Queensland (approval numbers 54584 and 2018001225). Given the novelty of this topic, there was no existing data to form the basis for a sample calculation. The selection of patients for this cohort was conducted randomly from individuals between December 2018 and February 2024 attending the Herston Oral Health Centre, a large 170-chair dental hospital in Brisbane, Australia. Two experienced periodontists independently conducted a comprehensive full-mouth periodontal charting using the Florida Probe periodontal charting system (Florida Probe Corporation, Gainesville, USA) to assess bleeding on probing (BOP), plaque index (PI), and probing pocket depths (PPDs). The inclusion criteria for subjects were: i) age ≥ 18 years, ii) presence of ≥ 20 teeth (excluding third molars), iii) no periodontal treatment or antibiotic therapy within three months before the investigation and iv) no long-term use of anti-inflammatory drugs. The exclusion criteria were: i) metabolic bone diseases, ii) autoimmune disease, iii) uncontrolled diabetes or post-menopausal osteoporosis, iv) pregnancy, and v) smoking.

Participants’ periodontal health was defined following the new classification of periodontitis guidelines [1]: (a) healthy: no periodontal disease history; PPD < 3 mm; BOP < 15 % sites; (b) gingivitis: no periodontal pockets, PPD < 3 mm; BOP > 30 % sites; (c) stage III/IV periodontitis: > 30% of the sites with PPD ≥ 3 mm and BOP, at least five sites with PPD ≥ 5mm on at least three non-adjacent teeth. The clinical characteristics of the participants are shown in **S.** **Table 1**. A total of 23 healthy participants, 14 gingivitis patients and 21 stage III/IV periodontitis patients were recruited with written consent. We compared salivary circRNAs profiles between: a) healthy, gingivitis and periodontitis groups (**Figure 1**); b) non-periodontitis (combining healthy and gingivitis) and periodontitis (**Figure 1**); and c) between Grade B and Grade C Periodontitis (**Figure 2**). Grading was carried out based on the assessment of radiographic bone loss (RBL) at the worst-affected tooth in the dentition as a function of age (Grade B: 0.25 ≤ RBL/age ≤ 1.0; Grade C: RBL/age > 1.0).

| S. Table 1*:* Participant demographics and clinical details | | | | |
| --- | --- | --- | --- | --- |
|  |  | **Healthy (n=23)** | **Gingivitis (n=14)** | **Periodontitis (n=21)** |
| Gender  n (%) | M | 12 (52.2%) | 10 (71.4%) | 16 (76.2%) |
|  | F | 11 (47.8%) | 4 (28.6%) | 5 (23.8%)  p=0.21 |
| Age |  | 52 ± 14.91  (24 - 75) | 42.57 ± 17.59  (22 - 65)  p=0.3053 | 62.57 ± 16.14  (41 – 88)  p=0.1663 |
| Ethnicity  n (%) | Caucasian | 19 (82.6%) | 7 (50%) | 17(80.9%) |
|  | Asians | 2 (8.7%) | 6 (42.86%) | 4 (19.05%) |
|  | Others | 2 (8.7%) | 1 (7.14%) | p=0.08 |
| BOP |  | 10 ± 4.18  (1% – 15%) | 45.29 ± 20.01  (30% - 100%)  * p <0.0001 | 28.33 ± 18.9  (10% - 55%)  *p<0.0001 |
| PI |  | 14.96 ± 10.99  (4% - 45%) | 33.43 ± 34.09  (0% - 100%)  * p=0.0406 | 27.81 ± 16.83  (10% - 55%)  * p=0.0186 |
| No. of deep pockets (>5 mm) |  | 0 | 0 | 29 ± 19.18  (5 - 66) |
| Average PPD (mm) |  | 2.22 ± 0.28 | 2.49 ± 0.36  * p=0.61 | 4.23 ± 1.32  * p<0.0001 |
| Periodontitis classification | Grade B |  |  | 12 (57.14%) |
|  | Grade C |  |  | 9 (42.86%) |
|  | Stage III |  |  | 13 (61.9%) |
|  | Stage IV |  |  | 8 (38.1%) |

**Abbreviations**: BOP: bleeding on probing; PPD: periodontal probing depth; PI: Plaque index. Age, BOP, PI, number of deep pockets, and average PPD are presented as mean ± SD (minimum–maximum) and were compared among groups using the Kruskal–Wallis test. Age and gender were analysed using the chi-square test. *: significant difference vs healthy control group

**Whole unstimulated saliva sampling**

Whole unstimulated saliva samples were collected before the full-mouth periodontal charting. The participants were asked to refrain from food and drink for at least 1 hour before sample collection. Between 9 am and 12 pm, the participants were asked to rinse their mouths to remove any food debris using 10 mL of water before saliva collection by the spitting method. The whole saliva (~2 mL) was collected by spitting into a sterile Falcon tube, and the samples were kept on ice before being aliquoted and frozen in a −80 °C freezer.

**Divergent Primer design for detecting circular RNAs**

Since most circRNAs are expressed from known protein-coding genes and consist of a single exon or of multiple exons [2], we chose ten mature circRNAs from ten known genes associated with periodontitis pathogenesis, including bone-turnover markers (alkaline phosphatase - ALP and runt-related transcription factor 2-RUNX2), Wnt pathway regulators (glycogen synthase kinase 3β- GSK3β; β-catenin - CTNNB and Wnt family member 5A - WNT5A) and Matrix metalloproteinase (MMP 9, MMP 16 and MMP17). The 10 circRNAs (listed in S Table 2) were *hsa_circ_0111987, hsa_circ_0003563, hsa_circ_0066893, hsa_circ_0064947, hsa_circ_0107474, hsa_circ_0066212, hsa_circ_0001161, hsa_circ_0001162, hsa_circ_0137250 and hsa_circ_0029447*.

| S. Table 2. Divergent primers used in this study | | | | |
| --- | --- | --- | --- | --- |
| Genes name | **Location** | **Gene and genomic length** | **Forward primer (5'-3')** | **Reverse primer (5'-3')** |
| *hsa_circ_0111987* | chr1:21880470-21890709 | ALP, 10239 bp | GTAAGGACATCGCCTACCAGC | GAGATGCAATCGACGTGGG |
| *hsa_circ_0003563* | chr6:45399599-45405788 | RUNX2,  6189 bp | CTTCACAAATCCTCCCCAAG | TTCCCGAGGTCCATCTACTG |
| *hsa_circ_0066893* | chr3:119585436-119595355 | GSK3B,9919 bp | AGGATTCGTCAGGAACAGGA | TCCCTTGTTGGAGTTCCCAG |
| *hsa_circ_0064947* | chr3:41278078-41278200 | CTNNB,  122 bp | TGGCTTGGAATGAGCGACAT | ATCTTGTGGCTTGTCCTCAGA |
| *hsa_circ_0107474* | chr17:63537572-63545778 | CTNNB,  8206 bp | TCCTCCTTATCGTGTGGGCA | CCTCTTGAAGGACCGGGAAA |
| *hsa_circ_0066212* | chr3:55514812-55521331 | WNT5A,  6519 bp | CGCCCAGGTTGTAATTGAAGC | GGGGCGCAACTAGGGAG |
| *hsa_circ_0001161* | chr20:44640275-44640841 | MMP9  566 bp | GTCTTCCCCTTCACTTTCCTGGG | CAGCGGTAGCCGTCGGAG |
| *hsa_circ_0001162* | chr20:44643022-44645125 | MMP9,  2103 bp | GTGCCATGTAAATCCCCACT | CTCCACTCCTCCCTTTCCTC |
| *hsa_circ_0137250* | chr8:89140544-89140653 | MMP16,  109 bp | CAATTGCTGTGTAAGTTACCCTT | TGGCCCTAATTTTCCCTACAGT |
| *hsa_circ_0029447* | chr12:132329662-132329994 | MMP17,  332 bp | TTTAAAGGCCCAGGAAGGACG | GAAAGCTTCACCCCGGATCT |
| *18S rRNA* |  |  | TTCGGAACTGAGGCCATGAT | CGAACCTCCGACTTCGTTC |
| *GAPDH* |  |  | AAACTGGAACGGTGAAGGTG | AGTGGGGTGGCTTTTAGGAT |
| *ACTB* |  |  | TCAGCAATGCATCCTGCAC | TCTGGGTGGCAGTGGC |
| Abbreviations: 18S rRNA: 18S ribosomal RNA; GAPDH: Glyceraldehyde 3-phosphate dehydrogenase; ACTB: β-actin. | | | | |

The design of divergent primers was carried out by CircInteractome (S. Figure 1) [3], targeting the expression of circRNAs originating from exons 3 and 4 of pre-mRNAs. To ensure specific amplification of circRNAs during reverse transcription (RT) followed by real-time quantitative polymerase chain reaction (qPCR) analysis, divergent primers spanning 100 bp were strategically designed across the back splice junction. Table 1 provides a list of the divergent primers designed for 10 chosen circRNAs.

**
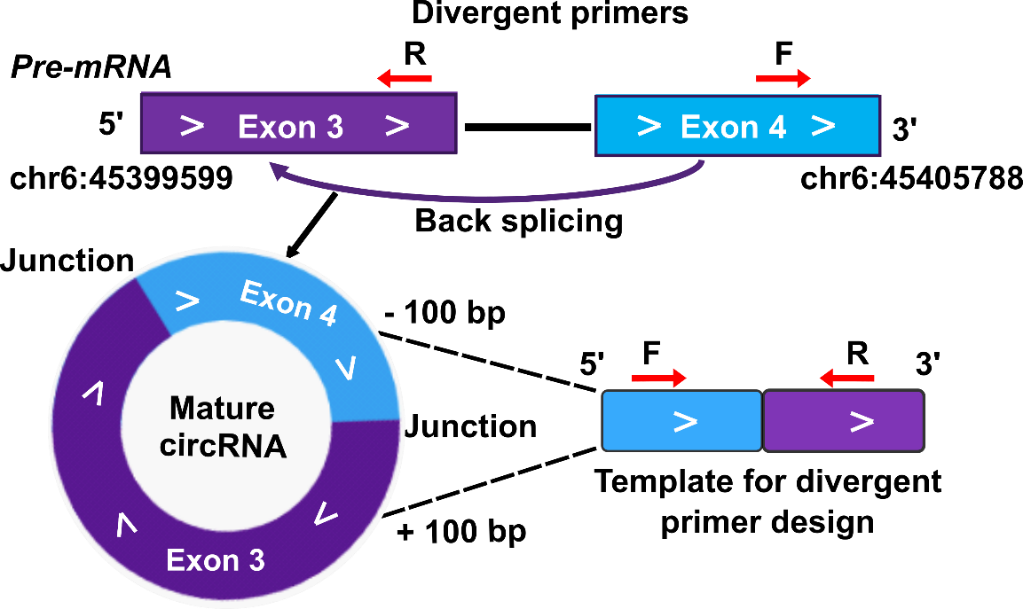
**

**S. Figure 1.** Schematic presentation of circRNAs biogenesis from back-slicing of pre-mRNA and divergent primer design using the junction as a template for RT-qPCR analysis. F: forward primer, R: reverse primer.

**Salivary circular RNA detection using RT-qPCR**

Salivary circRNAs were detected using divergent primers by RT-qPCR analysis as described previously [2, 4]. Total RNA was isolated from 300 µL whole saliva samples using Trizol reagent according to the manufacturer’s instructions and our published protocol [5, 6], with a slight modification. After the chloroform step, the aqueous phase containing RNA was mixed with 10% volume of sodium acetate (3M, pH 5.2) and 2.5 times 100% molecular-grade ethanol and incubated at -80^o^C overnight to precipitate the RNA. Following this, Ribonuclease R (RNase R, Lucigen, Astral Scientific) and PureLink DNase (ThermoFisher Scientific) were used to degrade linear RNAs and genomic DNA, respectively. Enriched circRNAs were subsequently isolated by a second round of RNA isolation [4].

The quantification of RNA was performed by Qubit™ RNA HS Assay Kit (Invitrogen) and NanoDrop™ One spectrophotometry (Thermo Fisher Scientific, Waltham, MA, USA), with 10 times more in Nanodrop than in Qubit (S. Figure 2).

**
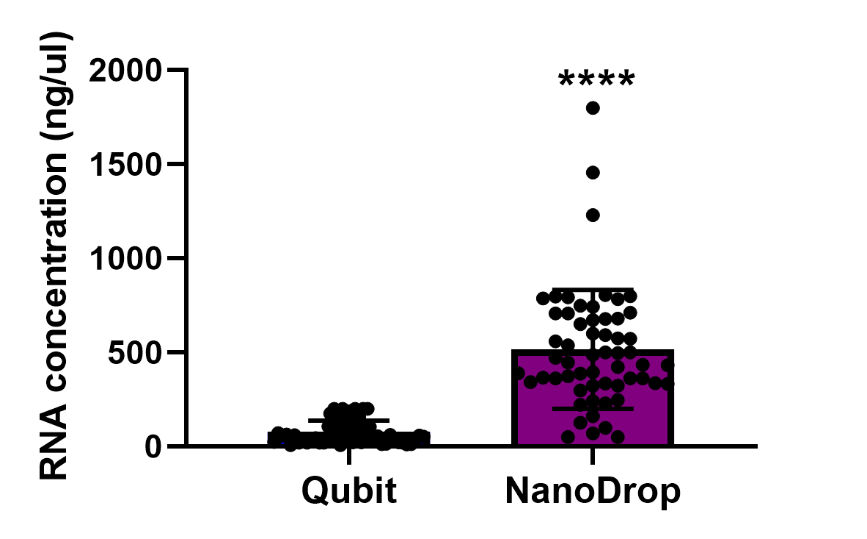
**

**S. Figure 2**. Different methods to quantify RNA quantity using Qubit and nanodrop, where Nanodrop led to ~10 times higher RNA yield than that from Qubit.

All enriched circRNAs were reverse-transcribed into cDNA with random primers using a First Strand cDNA Synthesis Kit (ThermoFisher Scientific, Scoresby, Australia) following the manufacturer’s instructions. Nested qPCR was performed using PowerUp™ SYBR™ Green Master Mix (ThermoFisher Scientific, Scoresby, Australia) in an ABI StepOnePlus instrument (Applied Biosystems, Scoresby, Australia) as described previously [7]. The first qPCR reaction was performed with 30 cycles with the divergent primers listed in Table 1, and then 1µL of the first qPCR product was used as a template for a second qPCR. All qRT-PCR experiments were performed in triplicate. RT-qPCR data were analysed by the ΔCt method and normalised to three housekeeping genes (β-actin, 18S rRNA and GAPDH) using the following formula: 2 ^−(normalized average Cts)^.

**Construction of circRNA–miRNA–mRNA Regulatory Network and Functional Enrichment Analysis via GO and KEGG**

To predict the potential functional networks and enrichment of differentially expressed circRNAs (*hsa_circ_0003563 and hsa_circ_0001161)*, CircNetVis—an interactive web application for visualising circular RNA interaction networks—was utilised [8]. The tool enabled visualisation and analysis of the interactions between circRNAs, microRNAs, mRNAs, and RNA-binding proteins, facilitating the identification of potential circRNA targets. <https://www.meb.ki.se/shiny/truvu/CircNetVis/>. Furthermore, Gene Ontology (GO) and Kyoto Encyclopedia of Genes and Genomes (KEGG) pathway enrichment analyses were performed for the miRNA target genes using CircNetVis. The results were visualised to highlight the most significantly enriched biological functions and pathways, where the X-axis represents statistical significance (–log10(p-value)), and the Y-axis displays the names of the top enriched GO terms and KEGG pathways.

**Statistical Analysis**

All data are displayed as mean ± standard deviation (SD), and statistical analysis was carried out using GraphPad Prism 10.0.0 software (San Diego, USA). Gender and ethnicity data were analysed using the chi-square test. A non-parametric Kruskal-Wallis using a Dunn’s multiple comparisons test was applied for data from healthy, gingivitis and periodontitis patients. A non-parametric Mann-Whitney unpaired t-test was used to analyse non-periodontitis vs periodontitis, and Grade B vs Grade C periodontitis data.

To determine the diagnostic ability of salivary circRNAs, Receiver Operating Characteristic (ROC) curves and the area under the curve (AUC) were used to measure the discriminatory power of the upregulated circRNAs as biomarkers for periodontitis using GraphPad Prism 10.0.0 as described previously [5, 9]. ROC curves and AUC were determined by the Wilson/Brown method between healthy controls and gingivitis and periodontitis, respectively. In all cases, a p-value < 0.05 was considered statistically significant. Differently expressed circRNAs as diagnostic biomarkers for periodontitis. We generated an ROC curve and AUC values to compare healthy vs gingivitis, healthy vs periodontitis, gingivitis vs periodontitis, non-periodontitis vs periodontitis and Grade B vs Grade C periodontitis.

**References**

1. Tonetti, M.S., H. Greenwell, and K.S. Kornman, *Staging and grading of periodontitis: Framework and proposal of a new classification and case definition.* Journal of Clinical Periodontology, 2018. **45**(S20): p. S149-S161.

2. Kristensen, L.S., et al., *The biogenesis, biology and characterization of circular RNAs.* Nature Reviews Genetics, 2019. **20**(11): p. 675-691.

3. Dudekula, D.B., et al., *CircInteractome: A web tool for exploring circular RNAs and their interacting proteins and microRNAs.* RNA Biol, 2016. **13**(1): p. 34-42.

4. Panda, A.C. and M. Gorospe, *Detection and Analysis of Circular RNAs by RT-PCR.* Bio-protocol, 2018. **8**(6): p. e2775.

5. Han, P., et al., *Salivary Small Extracellular Vesicles Associated miRNAs in Periodontal Status-A Pilot Study.* Int J Mol Sci, 2020. **21**(8).

6. Han, P. and S. Ivanovski, *Effect of Saliva Collection Methods on the Detection of Periodontium-Related Genetic and Epigenetic Biomarkers-A Pilot Study.* Int J Mol Sci, 2019. **20**(19).

7. Yoshimoto, R., et al., *Biosynthesis of Circular RNA ciRS-7/CDR1as Is Mediated by Mammalian-wide Interspersed Repeats.* iScience, 2020. **23**(7): p. 101345.

8. Nguyen, T.H., H.N. Nguyen, and T.N. Vu, *CircNetVis: an interactive web application for visualizing interaction networks of circular RNAs.* BMC Bioinformatics, 2024. **25**(1): p. 31.

9. Han, P., et al., *Salivary Outer Membrane Vesicles and DNA Methylation of Small Extracellular Vesicles as Biomarkers for Periodontal Status: A Pilot Study.* Int J Mol Sci, 2021. **22**(5).
